# Problematic versus reflective use: Types of social media use as determinants of mental health among young Filipino undergraduates

**DOI:** 10.34172/hpp.2022.11

**Published:** 2022-05-29

**Authors:** Jerome Visperas Cleofas, Julienne Celina Sicat Dayrit, Blulean Terosa Albao

**Affiliations:** ^1^Department of Sociology and Behavioral Sciences, De La Salle University, Manila, Philippines; ^2^Department of Literature, De La Salle University, Manila, Philippines

**Keywords:** COVID-19, Cross-sectional studies, Mental health, Sexual and gender minorities, Social media, Young adult

## Abstract

**Background:** The link between problematic social media use (SMU) and mental health among youth has been established. However, there is insufficient information on how mental health is influenced by COVID-19 pandemic-related changes and positive aspects of SMU. This study aims to determine the relationship of pandemic-related changes in SMU, and two types of SMU (problematic and reflective use) with mental health among young Filipino undergraduates.

**Methods:** A total of 1087 Filipino undergraduates aged 18 to 30 years old participated in this cross-sectional study. Data collection via online survey was conducted in August 2021.

**Results:** Findings indicate the significant association between the perceived changes in SMU and mental health among respondents (*P* <0.001). In terms of type of use, results suggest that students who demonstrate lower problematic SMU (B=-0.608, *P* <0.001, 95% CI=-0.955 – -0.259) and higher reflective SMU (B=3.524, *P* <0.001, 95% CI=2.051– 4.895) had higher mental wellbeing. Moreover, poorer mental wellbeing was observed among females and LGBTQ+ respondents with poorer internet quality (*P* <0.05).

**Conclusion:** Mental health among young Filipino undergraduates is influenced by pandemic-related changes and types of SMU. With the increasing necessity of social media amid the COVID-19 pandemic, mental health practitioners and advocates can increase their visibility online to promote reflective SMU as a protective factor against mental health decline.

## Introduction

Majority of social media users are young people.^1,2^ During the 2019 coronavirus disease (COVID-19) pandemic, the ubiquity of social media has become more profound due to the need for social distancing.^1,3,4^ Evidence has linked social media use (SMU) to poor mental health outcomes among the youth.^4-10^ While most of these studies focus on problematic or disordered SMU, there is reason to suspect that social media can also be used positively to cope with the challenges of the pandemic.^11^ There is limited research on the effect of more positive SMU on mental health.^6^

This study draws its focus on Filipino youth whose SMU increased during the pandemic.^12^ Evidence also demonstrated increased psychological challenges among Filipino youth during the same period.^13-17^ More research is needed to see whether these pandemic-related changes in SMU and mental health are associated, especially in Southeast Asian populations who are underrepresented in this area of study.^10,18^

This study appeals to media effects theory which posits that the deliberate or non-deliberate use of media (i.e. social media) can influence various psychological outcomes.^19,20^ Based on previous research that indicate parallelisms in the prevalence of SMU and mental health challenges before^21-23^ and during the pandemic,^24,25^ we hypothesize that *perceived pandemic-related changes in social media use and mental health status before and during the pandemic are significantly associated* (H1).

Aside from the frequency, we also aim to determine the relationship of the type of SMU on mental wellbeing. As indicated by pre-pandemic studies,^5,18,26-29^ we hypothesize that among Filipino young undergraduates*, problematic SMU will significantly negatively predict mental wellbeing* (H2). In terms of more positive use of social media, scholars have suggested that using social media reflectively have psychological benefits.^30-33^ Hence, we hypothesize that *reflective SMU will significantly positively predict mental wellbeing* (H3).

## Materials and Methods

### Study design and sampling

This study is part of a larger independent research project that examined the various social media behavioral and health outcomes during the pandemic among young Filipino undergraduates. Specifically, this present study made use of a cross-sectional design. The eligibility criteria for this study are college students enrolled and residing in the Philippines, aged 18 to 30 years old, which is the “youth” bracket based on the National Youth Commission.^34^ Our recruitment was done via social media (Facebook and Twitter), targeting accounts that were geolocated in the Philippines within the youth age bracket. Based on G*Power analysis (power = 0.95; effect size = 0.15), the minimum sample required is 211. The total number of students who responded to the survey was 1152; however, after the survey was rid of ineligible entries, the final sample that fit the criteria was trimmed down to 1087, which is five times more than the required number of respondents. Majority of the respondents resided in North and Central Luzon (50.5%), followed by the National Capital Region and South Luzon (37.90%), while the rest came from Visayas-Mindanao.

### Instruments

*Demographic characteristics.*We collected the following demographic data from our respondents: age (in years), sex assigned at birth (male = 1, female = 0), sexual orientation and gender identity (SOGI) and estimated household income (based on the brackets suggested by Philippine Institute for Development Studies). For SOGI, those who answered homosexual, bisexual, queer and asexual in sexual orientation, and/or transgender in gender identity, including who answered “prefer not to disclose” were assigned under LGBTQ+ ( = 0). Others were categorized as cisheterosexual ( = 1). Demographic variables were used as covariates in the hierarchal regression.

*Digital profile.*Three sub variables were included under digital profile. First is number of social media sites wherein which they have an active account. Second is the number of types of gadgets they owned. We provided the respondents checklists containing common social media sites (e.g. Facebook, Twitter, Instagram, YouTube, etc.) and computing devices (e.g. smartphone, laptop, desktop, etc). They were provided a blank to indicate other sites and gadgets they had that were not part of the list. The third sub variable is perceived Internet quality, measured through a 7-point Likert scale (1 = very bad; 7 = very good). We asked, “*How would you rate the reliability of your internet connection?*” Demographic characteristics and digital profile were used as covariates in this study. Digital profile variables were used as covariates in the hierarchal regression.

Perceived pandemic-related change in SMU was measured by asking the respondents the select one among four statements: “I used social media most frequently before the pandemic” (pre-pandemic) “I used social media most frequently in 2020, during the first year of the pandemic” ( < 2020), “I used social media most frequently in 2021, during the second year of the pandemic” ( < 2021). “My social media use has NOT changed since pre-pandemic” (no change).

*Perceived pandemic-related change in mental health status* was measured by instructing the respondents to choose among the following four statements: “*My mental health was worst before the pandemic*” (pre-pandemic), “*My mental health was worst in 2020, during the first year of the pandemic*” ( < 2021), “*My mental health has been the worst in 2021, during the second year of the pandemic*” ( < 2021), and “*My mental health has NOT changed since pre-pandemic*” (no change).

To measure problematic SMU, the Social Media Disorder-Short Form scale (SMD-SF) by van den Eijnden et al.^35^ was used. SMD-SF is a 9-item scale that measures preoccupation, tolerance, withdrawal, persistence, displacement, problem, deception, escape and conflict in the use of social media. It is measured as a “Yes” or “No” question for each item. This scale has been found to have an acceptable factor structure, intra-class correlation coefficient (0.663, 95% CI:0.565-0.739) and internal consistency (Cronbach alpha = 0.82). Sample question is “*during the past year, have you felt the need to use social media more and more often?*”

To measure reflective SMU, the anticipatory reflection component of the Social Media Competency Scale for College Students (SMCS-CS) by Zhu et al.^30^ was used. This adopted scale has 9-items, inquiring their level of agreement (1 = strongly disagree, 5-strongly agree) on certain statements about the extent to which they practice reflection in the use of social media. A sample statement is “*I would consider the possible consequences before using social media to write something*.” The Cronbach alpha score of the scale is 0.95.

Mental wellbeing, the outcome variable of the regression model, was measured using the 14-item Warwick-Edinburgh Mental Wellbeing Scale (WEMWBS) by Health Scotland, University of Warwick, and University of Edinburgh.^36^ A sample item is “*I’ve been feeling good about myself*.” Possible responses range from 1 (none of the time) and 5 (all of the time). The short version of the scale has an acceptable of reliability (a = 0.87) in the Filipino adult population.^37^

### Data gathering procedure and ethical considerations

After securing ethical clearance from our host department in the university, we began our data collection via social media. We created a Facebook Page where we posted the link to our online survey form. Included with the link, we posted the details on the research objectives, procedures, and participant requirements. We made use of Facebook Advertisement services to boost the link’s engagement with accounts geolocated in the Philippines. We also made use of our social networks on Facebook and Twitter to recruit more participants. The data collection period was during the first three weeks of August 2021.

We secured informed consent digitally, through the first page of the google form, where the study’s details and ethical considerations were presented. Respondents who clicked “YES” would be brought to the survey proper. No personal or private information were collected. All data gathered from the study were anonymized and secured in a password-protected cloud storage.

### Data analysis procedure

The descriptive statistics we used were frequency and percentage for categorical variables, and mean and standard deviation for continuous variables. To test the association between perceived changes in SMU and mental health (H_1_), chi-square (contingency tables) was used. To examine the relationship between demographic characteristics, digital profile, and types of SMU, and mental wellbeing (H_2_ and H_3_), bivariate (independent t-test and Pearson R correlation) and multivariate (hierarchal regression test) were used. The dependent variable is mental wellbeing (WEMWBS). The explanatory variables for the first step were problematic and reflective SMU, then the covariates based on the significant correlates from sociodemographic and digital use profiles were added for the second step. Bootstrapping (n = 5000) was applied in the regression analysis, in order to address any possible issues of non-normality by presenting bias corrections based on simulated data.^38^ Significance was set at α = 0.05. JASP 0.14.1^39^ was used for the analysis.

## Results

### Descriptive results

Table 1 shows the descriptive statistics of the key variables of the study. Majority of the participants are 21 years old (mean = 20.1, SD = 1.73), female (n = 661, 60.81%), cisheterosexual (n = 822, 75.62%), with estimated family income of PhP 10 956.00 and below (n = 375, 34.50%). Moreover, most student respondents report having active accounts in 5 social media sites (mean = 4.02, SD = 1.67), two different gadget types (mean = 1.98, SD = 0.95) and an above average internet quality (mean = 4.75, SD = 1.20 out of 7).

**Table 1:** Descriptive statistics (N = 1087)

**Variables**	**n/Mean**	**%/SD**
Age	20.1	1.73
Gender		
Male	426	39.19
Female	661	60.81
Sexual Orientation and Gender Identity (SOGI)		
Cisheterosexual	822	75.62
LGBTQ+	265	24.48
Family Income Bracket		
PhP 219 140 and above	75	6.90
PhP 131 483 to P219 140	60	5.52
PhP 43 828 to P76 668	110	10.12
PhP 76 669 to P131 484	92	8.46
PhP 21 914 to P43 827	164	15.09
PhP 10 957 to P21 913	211	19.41
PhP 10 956 and below	375	34.50
Number of social media sites (range = 1 to 12)	4.02	1.67
Number of gadgets types owned (range = 1 to 7)	1.98	0.95
Perceived internet quality (range = 1 to 7)	4.75	1.20
Pandemic-related Changes in Social Media Use		
Social media use highest pre-pandemic	163	15.00
No change reported	311	28.61
Social media use highest in year 2020	352	32.38
Social media use highest in year 2021	261	24.01
Problematic social media use (range = 1 to 9)	3.23	2.38
Reflective social media use (range = 1 to 5)	3.79	0.65
Pandemic-related changes in mental health status		
Mental health worst pre-pandemic	87	8.00
No change reported	220	20.24
Mental health worst in year 2020	417	38.36
Mental health worst in year 2021	363	33.40
Mental wellbeing	43.6	12.9

In terms of pandemic-related changes in SMU, majority of the respondents reported the highest use of social media in 2020, during the first year of the pandemic (n = 352, 32.38%). In addition, the students demonstrated low levels of problematic SMU (mean = 3.23, SD = 2.38 out of 9) and above average levels of reflective SMU (mean = 3.79, SD = 0.65 out of 5).

As for mental wellbeing, most respondents reported the worst mental health also in 2020 (n = 417, 38.36%). Meanwhile, the findings indicate a moderate level of subjective mental wellbeing (mean = 43.6, SD = 12.9) during the time of data collection.

### Association between perceived pandemic-related changes in social media use and mental health status

**Figure 1:**
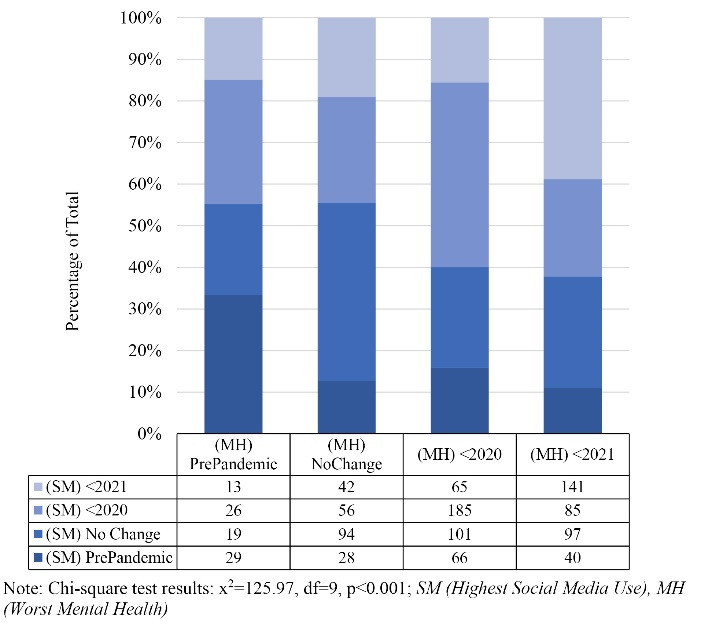
Association between reported pandemic-related changes in social media use and mental health status (N = 1,087)

Figure 1 indicates the significant association between reported pandemic-related changes in SMU and mental health status among the students based on chi-square results (*P* < 0.001). The graph and contingency tables suggest that the highest proportion of respondents reporting the worst mental health in each time period, also report the highest SMU during the same time period (pre-pandemic [n = 29, 33.33%], year 2020 [n = 185, 44.37%], year 2021 [n = 141, 38.84%]). Additionally, the largest group among those reporting no changes in mental health from pre-pandemic to 2021 levels also reported no changes in SMU (n = 94, 42.72%).

### Bivariate statistics for type of profile, type of SMU and mental wellbeing

Table 2 suggests that age (r = 0.065, *P* = 0.033), gender (t = 2.45, *P* = 0.014), SOGI (*P* = 0.001), perceived internet quality (r = 0.177, *P* < 0.001), problematic SMU (r = -0.138, *P* < 0.001), reflective SMU (r = 0.203, *P* < 0.001) were significantly correlated with subjective mental wellbeing. Higher levels of subjective mental wellbeing were observed among older, cisheterosexual males who have access to better internet quality, and reporting lower problematic SMU and higher reflective SMU. Income, number of social media sites and gadget types owned did not yield significant *P* values.

**Table 2:** Mental wellbeing and its relationship with demographic and digital profile and problematic and reflective social media use

**Variables**	**Mean±SD**	***P*** ** value**
Age	—	0.033
Gender		
Male	44.8 ± 12.6	0.014
Female	42.8 ± 13.0	
SOGI		
Cisheterosexual	44.3 ± 12.7	0.001
LGBTQ+	41.4 ± 13.3	
Family Income Bracket		
PhP 219 140 and above	44.3 ± 11.5	0.984
PhP 131 483 to P219 140	43.8 ± 12.8	
PhP 43 828 to P76 668	43.7 ± 13.9	
PhP 76 669 to P131 484	44.2 ± 12.5	
PhP 21 914 to P43 827	43.6 ± 12.2	
PhP 10 957 to P21 913	43.7 ± 12.2	
PhP 10 956 and below	43.1 ± 13.7	
Number of social media sites	—	0.196
Number of gadgets types owned	—	0.735
Perceived internet quality	—	< 0.001
Problematic social media use	—	< 0.001
Reflective social media use	—	< 0.001

Note: Pearson R correlation used for continuous variables, independent *t* test for dichotomous variables (gender, SOGI); one-way ANOVA for categorical variables (family income bracket)

### Hierarchal regression results for type of SMU and mental wellbeing

Table 3 presents the results of the bootstrapped (n = 5000) hierarchal regression analyses to identify the significant predictors of subjective mental wellbeing. Durbin-Watson statistics ranged from 2.048 to 2.040, suggesting no autocorrelation in the sample. In step 1, the types of SMU explained 5.4% of the variance of subjective wellbeing (F = 32.268, *P* < 0.001). Problematic SMU (B = -0.664, *P* < 0.001, 95% CI = -1.016 – -0.320) and reflective SMU (B = 3.840, *P* < 0.001, 95% CI = 2.299–5.129) significantly predicted subjective mental wellbeing. The direction of problematic SMU is negative, and reflective SMU is positive.

**Table 3:** Hierarchal regression test for predictors of subjective mental wellbeing

**Variables**	**Step 1**	**Step 2**
Block 1: types of social media use		
Problematic social media use	-0.664***	-0.608***
Reflective social media use	3.840***	3.524***
Block 2: profile variables		
Age		0.406
Gender (Male = 1)		2.331**
SOGI (Cisheterosexual = 1)		2.648**
Perceived internet quality		1.614***
Overall Model		
F	32.268***	19.302***
R^2^	0.054	0.092
∆R^2^		0.038
Durbin-Watson Statistic	2.048	2.040

Note: *P < 0.05, ** P < 0.01, *** P < 0.001; Values represent unstandardized estimates. Bootstrapping based on 5000 replicates.

In step 2, the significant demographic and digital correlates were included in the regression equation. The model explained 9.2% of the variance of subjective mental wellbeing (F = 19.302, *P* < 0.001). The R^2^ change from step 1 to step 2 was 3.8%. When profile variables were added into the model, the predictive relationship of problematic SMU (B = -0.608, *P* < 0.001, 95%CI = -0.955 – -0.259) and reflective SMU (B = 3.524, *P* < 0.001, 95% CI = 2.051–4.895) with subjective mental wellbeing remained significant. Moreover, gender (B = 2.331, *P* < 0.002, 95% CI = 0.846–3.819), SOGI (B = 2.648, *P* = 0.003, 95% CI = 0.795-4.386) and perceived internet quality (B = 1.614, *P* < 0.001, 95% CI = 0.977–2.223) were significant predictors of subjective mental wellbeing. Age was not a significant predictor.

## Discussion

This study examined pandemic-related changes in SMU, and types of SMU (problematic versus reflective) as determinants of mental health among young Filipino undergraduates. Our findings indicated the significant association between pandemic-related changes in SMU and mental health, confirming our first hypothesis. Most of the students who noted the worst mental health status in each time period also reported the highest SMU during the same period. This result corroborates with previous studies elsewhere that have linked the higher levels of exposure to social media with poor mental health outcomes before^27,40,41^ and during the pandemic.^7,21,23-25,42^ Moreover, we note a higher concentration of respondents reporting the highest level of SMU and worst mental health during the first year of the pandemic versus the second year (2020 > 2021). The decrease of SMU and the improvement of mental status of the respondents may have been influenced by the gradual easing of lockdown measures, similar to what was noted in a previous longitudinal study in Austria.^43^

Our results for the second objective suggest that both types of SMU significantly contributed to the mental wellbeing of the respondents. As hypothesized, the two types of SMU demonstrated opposing directions. Consistent with evidence before^5,26^ and during the pandemic,^7,29^ our current study demonstrates the inverse relationship between problematic SMU and mental wellbeing. During the time of COVID-19, individuals had been forced to use social media for work, study, and other social activities due to pandemic-induced confinement^44^; this increased dependence on social media may have triggered its negative effect on mental health.^45^ Moreover, social media detoxification arguably related to COVID-19 information avoidance has been linked to positive psychological outcomes among youth in the Philippines.^46^

On the other hand, our findings indicate that reflective SMU significantly positively predicted mental wellbeing. This confirms the theoretical assertion of Zahrai et al^31^ that the role of reflective psychological processes in protecting individuals from the ill effects of social media. Similarly, Maheux et al^47^ suggested that during the pandemic, the meaningful use of social media can foster a sense of gratitude, and consequently, improve psychological wellbeing. Overall, the inferential findings of our study extend one of the tenets of media effects theory, which posits that media effects are conditional.^19^ The effect of media on mental health outcomes can be shaped by social context (i.e. COVID-19 pandemic) and individual behavioral differences (i.e. type of SMU).

Additionally, gender and SOGI emerged as significant predictors of subjective mental wellbeing. Consistent with previous studies among Filipinos,^13,14,16^ female undergraduates reported poorer mental wellbeing compared to their male counterparts. Moreover, LGBTQ+ students demonstrated lower mental wellbeing compared to their cisheterosexual counterparts, which corroborates with previous evidence among LGBTQ+ emerging adults in the United States that indicate decreased hope, social connections, and pride during the pandemic.^48^ Some gender issues in the Philippines that might have contributed to the lower mental health include inequity in access to basic social and health services, increased incidence of gender-based violence and disruption of sexual and reproductive health services.^49^ Finally, findings indicate that better perceived Internet quality was significantly linked to undergraduates’ mental wellbeing. The transition of education and other facets of social life online place Filipino college students with better type and duration of Internet connectivity can protect against COVID-19 anxiety, as seen in previous research.^14,16^

### Strengths and limitations of the study

To our knowledge, this is the first study that attempted to measure changes in SMU and mental health in different moments pre- and during COVID-19 pandemic. Moreover, this is the first research that examined not only problematic SMU, but also reflective SMU as factors to mental wellbeing. However, despite its novelty and large sample size, the present study is limited by its sampling design, which recruited participants via social networking sites, thus constraining its generalizability. Moreover, the youth from the southern parts of the country were underrepresented. The analysis did not account for geographic location, which could have influenced their Internet and pandemic experience. It must also be noted that the measures of change in SMU and mental health are forced-single-item and perceptual in nature, which may not be able to accurately track the trends of the two variables of interest. Additionally, this study is cross-sectional in nature, thus causality of the relationships established cannot be ascertained. Also, our regression model had a relatively low explanatory power (9.2%); however, this is expected and is consistent with the estimates of recent meta-analytic evidence.^9^ Future research can replicate the protocol to a large, random, and representative sample. Researchers may also consider conducting a longitudinal study that will be able to objectively monitor problematic and reflective SMU, and measure more specific mental health states such as depression, anxiety, and loneliness.

## Conclusion

Our findings provide new evidence on how changes in SMU and mental health across time can run parallel with each other. However, the impact on social media on mental wellbeing among the youth depends on the way they use it: problematic use deters mental health, while reflective use improves mental wellbeing. Moreover, our results also highlight that females and LGBTQ+ persons, and those experiencing digital disadvantage experience poorer mental health.

Thus, it is important for teachers, school health practitioners and families to monitor the changes and behaviors of young undergraduates towards social media. Universities can employ learning activities that foster reflective SMU to take advantage of its beneficial effects on mental wellbeing. Health and helping professionals can empower families in detecting and addressing problematic SMU and mental health problems. Mental health practitioners and advocates can increase their visibility online to conduct psychoeducation regarding judicious use of social media towards better mental health. Furthermore, our current study highlights the salience of implementing mental health promotion and protection research, initiatives and programs that are inclusive to gender and sexual minorities. Community health and helping professionals must address possible cases of gender-based discrimination and violence that may be experienced by undergraduates who are studying while at home. Moreover, our present research emphasizes the importance of Internet accessibility to achieve better mental wellbeing during the pandemic, and underscores the urgency of creating policies and infrastructures that will improve the quality and expand the reach of Internet providers.

## Acknowledgments

We would like to extend our gratitude to the undergraduates who participated in this research.

## Authors’ Contributions

JVC: Study conceptualization, methodology, analysis, writing – original draft, writing – review and editing; JCSD: methodology, data acquisition, writing – original draft; BTA: methodology, data acquisition, writing – original draft.

## Funding

No funding was received for this study.

## Ethical approval

Administrative clearance for ethical conduct of the study was granted by the Department of Sociology and Behavioral Sciences of DLSU (2021-08-09). Informed consent was secured from all respondents. National and local policies on data privacy were observed during data collection and storage.

## Competing Interests

Authors declare that there is no conflict of interest.
